# Gaping with Cough

**Published:** 1897-05

**Authors:** 


Gaping with Cough.—*Anacardium orientale*—After the attacks of the whooping-cough a long, lasting gaping and drowsiness. Boenninghausen. Compare with *Opium*. Characteristic by Dr. C. Raue. Cough *after* gaping; or gaping increases the cough. *Arn.*, *Cin. Mur-ac.*, *Nux-vom.*, *Staph.* According to Boenninghausen, coughing *and* gaping before, after, and between the spells, have also: *Anac.* after the cough; *Ant-tart.*, gapes frequently; *Arnica* also after gaping.

---

* *Traité de la Pneumonie*, p. 517.

